# Association of Nuclear-Localized Nemo-Like Kinase with Heat-Shock Protein 27 Inhibits Apoptosis in Human Breast Cancer Cells

**DOI:** 10.1371/journal.pone.0096506

**Published:** 2014-05-09

**Authors:** Gina Shaw-Hallgren, Katarzyna Chmielarska Masoumi, Reihaneh Zarrizi, Ulf Hellman, Per Karlsson, Khalil Helou, Ramin Massoumi

**Affiliations:** 1 Translational Cancer Research, Molecular Tumor Pathology, Department of Laboratory Medicine, Lund University, Lund, Sweden; 2 Ludwig Institute for Cancer Research, Uppsala, Sweden; 3 Institute of Clinical Sciences, Department of Oncology, University of Gothenburg, Gothenburg, Sweden; Wayne State University, United States of America

## Abstract

Nemo-like kinase (NLK), a proline-directed serine/threonine kinase regulated by phosphorylation, can be localized in the cytosol or in the nucleus. Whether the localization of NLK can affect cell survival or cell apoptosis is yet to be disclosed. In the present study we found that NLK was mainly localized in the nuclei of breast cancer cells, in contrast to a cytosolic localization in non-cancerous breast epithelial cells. The nuclear localization of NLK was mediated through direct interaction with Heat shock protein 27 (HSP27) which further protected cancer cells from apoptosis. The present study provides evidence of a novel mechanism by which HSP27 recognizes NLK in the breast cancer cells and prevents NLK-mediated cell apoptosis.

## Introduction

MAP kinases are classified into conventional or atypical enzymes, based on their phosphorylation status and activation by MAP kinase kinase (MAPKK) family members. Nemo-like kinase (NLK) is a proline-directed serine/threonine kinase, belonging to the atypical MAP kinases, and is regulated by phosphorylation [1]. Homeodomain-interacting protein kinase (HIPK2) [2]–[5], MAPKKK TGF-β-activated kinase 1 (TAK1) [2], [6], [7], and p38 MAPK [8], are enzymes that have been suggested to activate NLK through phosphorylation. Upon activation, NLK can phosphorylate several proteins essential for the regulation of different signaling pathways, such as Wnt/β-catenin [6], [7], [9], [10], Notch [11], [12], and Smad [13].

NLK has been shown to negatively regulate Wnt/β-catenin signaling by phosphorylation of the complex LEF1/TCFs, which facilitates ubiquitination and degradation of this complex [7]. The ubiquitination of TCF/LEF is executed by NARF (NLK associated RING finger protein), acting as an E3 ligase [14]. In addition, β-catenin-induced transcriptional activation can be antagonized by NLK through activation of the TAK1-mediated non-canonical Wnt pathway [7]. It was recently shown that TAK1 activation of NLK does not occur through direct interaction, but TAB2 may scaffold the association between TAK1 and NLK [15], [16]. Furthermore, SETDB1 (SET domain bifurcated 1), a histone methyltransferase, is phosphorylated by NLK, upon Wnt5a stimulation. Phosphorylation of SETDB1 leads to disruption of the PPAR-gamma function through methylation, a mechanism shown to be vital for lineage decision of mesenchymal stem cells [15], [17], [18].

Besides Wnt, NLK was shown to antagonize Notch signaling during neurogenesis. NLK negatively regulated Notch-dependent transcriptional activation by phosphorylation of a member of the Notch-mediated transcriptional complex, NotchICD. The phosphorylation of NotchICD by NLK blocked its ability to form a transcriptionally active ternary complex [12]. C-Myb [2], [5], Smad4 [19], and STAT3 [20], [21] are other targets for phosphorylation by NLK. Serine phosphorylation of STAT3 is necessary for mesoderm induction [21], whereas phosphorylation of c-Myb promotes its proteasome-dependent degradation [3]–[5], [21]. FOXO1 [22] and myocyte enhancer factor 2A (MEF2) [23] are two recently identified transcription factors, regulated by NLK. The phosphorylation of FOXO1 by NLK inhibits its transcriptional activity through a nuclear export process [22], while phosphorylation of MEF2 by NLK is crucial for Xenopus laevis development [23]. NLK also contributes to the reorganization of the cytoskeleton. Phosphorylation of microtubule-associated protein-1B (MAP1B) and of the focal adhesion protein, paxillin, stimulates NGF-induced re-distribution of F-actin as well as neurite outgrowth [24].

The role of NLK in cancer is not well known. Induction of wildtype NLK in human colon carcinoma cells (DLD-1) was shown to trigger programmed cell death [25], [26]. This mechanism involved phosphorylation of CBP and consequential suppression of the transcriptional activity of AP-1, Smad, and p53, all of which use CBP as a co-activator [4], [26]. In prostate cancer, NLK expression was decreased at the mRNA level in the tumor site, but no significant differences in the NLK protein expression were observed. Furthermore, overexpression of NLK prompted a more effective induction of apoptosis in AR-expressing prostate cancer cells than in AR-negative cells [27]. However, although NLK was revealed to be overexpressed in hepatocellular carcinomas, depletion of NLK reduced cell growth, and did so by inhibiting the expression of cyclinD1 and CDK2, both essential for the mitogenic potential of tumor cells [28].

Recent studies reported that NLK can be localized in the cytosol or in the nucleus, and that homodimerization of NLK is essential for nuclear localization [29]. However, the impact of specific subcellular localization of NLK is not well established. The present paper discloses that NLK was localized mainly in the nuclei of breast cancer cells. Moreover, the association of NLK with HSP27, which was identified as a novel binding partner for NLK, protected the cancer cells from apoptosis.

## Materials and Methods

### Tumor material and ethical approval

Full-faced formalin-fixed, paraffin-embedded tumor and non-tumor tissues (FFPE) were obtained from the Department of Pathology at Sahlgrenska University Hospital in accordance with the Declaration of Helsinki. Our study is not a clinical trial and the tumor specimen were used anonymously therefore, patient consent is not needed and the research on these tumors is approved by the Medical Faculty Research Ethics Committee, Gothenburg, Sweden (s164-02). In addition, the review board waived the need for written informed consent from the participants. All samples were obtained from patients undergoing surgical resection in Gothenburg, Sweden, between 1990 and 2006. FFPE sections, 4 µm thick, were applied onto positively charged slides (FLEX IHC microscope slides, Dako, Sweden) for immunostaining, in order to assess NLK protein expression in tumor as well as adjacent normal breast tissue.

### Cell culture

All cell lines were purchased from the American Type Culture Collection (ATCC) and grown under standard conditions. Cells of human breast and breast cancer were routinely grown for 7 days at 37°C and 5% CO_2_ as follows: MCF10A cells were cultured in Dulbecco's modified Eagles medium/F12, supplemented with 5% horse serum (Sigma), 20 ng/ml EGF (Millipore), 100 ng/ml cholera toxin (Sigma), 0.01 mg/ml insulin (Sigma), 500 ng/ml hydrocortisone (Sigma), and 0.1% penicillin/streptomycin (GIBCO). BT549 cells were cultured in RPMI, supplemented with 10% FBS (Sigma), 0.1% penicillin/streptomycin (GIBCO). Cells of MCF7, CAMA1, and MDA231 were cultured in Dulbecco's modified Eagle's medium, supplemented with 10% FBS (Sigma) and 0.1% penicillin/streptomycin (GIBCO).

For DNA sequencing of breast cell lines we used the following amplifying (Ampl.) and sequencing (Seq.) primers:

Ampl prod K155 Forward: TGGCAGATGAGTTGGGTGAC

Ampl prod K155 Reverse: AACCTCACTGGTGCGCTTAT

Ampl prod T286 Forward: TCAAAGTTCAGTGCCCTATCAA

Ampl prod T286 Reverse: TCAATACTCCCTCTAAAGGCTCA

SeqPrim K155 F1: TGGAAAACAAGGTGTGGAACTG

SeqPrim K155 F2: CCCCTTCCCCAAATATTGCTT

SeqPrim T286 R1: TAATGACGGCTGCCCATCAG

SeqPrim T286 R2: GGGACTCTGTGCCTGAAACA

### Transient Transfection

Transient transfection assays were carried out in six-well plates at 80% confluence, using PolyFect Transfection Reagent (Qiagen) in accordance with the manufacturer's recommendations. Twenty four hours prior to transfection, cells were seeded in medium containing 10% FBS. Cells were incubated at 37°C and 5% CO_2_ for 24–48 hours. The medium was removed and the cells washed with PBS, after which fresh serum-containing medium was added to the cells. DNA, 1.5–4.0 µg, was mixed with an appropriate volume of OptiMEM (Sigma), followed by addition of 10–25 µl PolyFect transfection reagent. Samples were incubated at room temperature for 10 minutes, in order to allow complex formation to be completed, prior to being transferred to the cells. Cells were subsequently incubated for 24, 48, or 72 hours. For HSP27 or NLK knockdown, MCF7 cells were transfected once or two times with 50 nM HSP27-siRNA (Santa Cruz Biotechnology, Inc. [sc-29350]), HSP27-siRNA (Cell Signaling Technology, Inc. [SignalSilence #6356]), NLK shRNA Plasmid (Santa Cruz Biotechnology, Inc, [sc-36079-SH]), Control shRNA Plasmid (Santa Cruz Biotechnology, Inc, [sc-108060], or NLK-siRNA oligos (TAG Copenhagen, Denmark [NLK-Sense: GAA CCT CAG CTC TGT CCG ATT; NLK-Antisense: TCG GAC AGA GCT GAG GTT CTT]) using Lipofectamine 2000 (Invitrogen) in accordance with the supplier's recommendations.

### Antibodies

Antibodies to NLK (Abcam ab26050 and ab97642), Tubulin (Sigma T9026), β-actin (Sigma A5316), HSP27 (Santa Cruz [c-20] sc-1048), phospho LEF Thr155 (Millipore ABS485), total LEF (Thermo Scientific 01674297), Lamin B (Thermo Scientific MA1-5822), GST (GE Healthacare 27457701V), pHSP27 (Cell Signaling Technology 2401) and FLAG (Sigma A8592) were used. The HRP-conjugated secondary antibodies were from DAKO, and the Alexa Fluor fluorescent antibodies from Invitrogen.

### Cell lysis and fractionation

Cells were grown on 10 cm plates to 80% confluence, harvested in PBS, and lysed in buffer comprising 1 M TrisHCl (pH 7.6), 5 M NaCl, 0.5 M EDTA, 1% Triton X-100, and Complete protease inhibitor cocktail (Roche). Protein concentration was assessed using the Bradford method, and sample concentrations were adjusted accordingly. Lysates were boiled for 5 minutes at 100°C with a 2× loading buffer, containing 1 mM dithiothreitol (DTT).

For cell fractionation, cells were lysed with cytosolic lysis buffer, containing 10 mM Hepes (pH 7.9), 10 mM KCl, 0.1 mM EDTA, 1 mM DTT, and protease inhibitors, and the nuclei were subsequently harvested by centrifugation. Supernatants, containing the cytosolic fraction, were further purified through an additional centrifugation step. The nuclei were ruptured by treatment with nuclear extraction buffer (20 mM Hepes [pH 7.9], 0.4 M NaCl, 0.1 mM EDTA, 0.1 mM EGTA, 1 mM DTT, 1 mM PMSF and 0.1% NP40), and by passing 10 times through a 27G needle, followed by centrifugation to purify. A Bradford assay was performed, and both fractions were boiled with 2× loading buffer, containing 1 mM DTT.

### Immunoprecipitation and Western blotting

Cells were harvested and lysed for immunoprecipitation, as previously described [43]. Lysates were probed overnight with selected antibodies, and complexes were captured using Protein A Sepharose beads (Sigma) or Anti-FLAG M2 affinity gel (Sigma). The beads/gel were washed four times with lysis buffer, after which the protein complexes were extracted, either with FLAG peptide solution (Sigma), or through boiling with 2× loading buffer, containing 1 mM DTT. Immunoprecipitates were subjected to SDS-PAGE, and the resolved proteins were transferred to PVDF membranes (Amersham Biosciences). Western blotting was carried out, following standard protocol. The chemoluminescence was detected with a charge coupled device camera (Alpha Innotech).

### Immunofluorescence and confocal microscopy

Cells were grown on glass coverslips in 6-well plates for 24, 48, and 72 hours. After paraformaldehyde (PFA) fixation (4% for 15 minutes), cells were permeabilized with 0.25% Triton X-100 solution, after which they were blocked with 1% BSA, and subsequently probed with antibodies against NLK, FLAG, or HSP27. After washing the coverslips, fluorescent antibodies including Alexa 488 goat anti-rabbit and Alexa 568 donkey anti-goat obtained (Invitrogen Life technologies) were applied. Nuclei were stained with DAPI, and the coverslips were washed again, before mounting onto glass slides.

### MALDI-TOF mass spectrometry analysis

Immune complexes were obtained as described under the immunoprecipitation section. Iodoacetamide (100 mM) was added to the complexes, and the precipitated proteins were incubated for 20 minutes in darkness. The samples were separated by SDS-PAGE polyacrylamide gel (Invitrogen). The gel was silver-stained as previously described [30]; i.e. it was first washed for 20 minutes in 50% methanol with 5% acetic acid, and then placed in 50% methanol for 10 minutes, followed by sensitization with 0.02% sodium thiosulphate, prior to staining with 0.1% silver nitrate for 20 minutes, at room temperature. The gel was developed for less than 10 minutes in a solution of 0.04% formalin and 2% sodium carbonate, followed by 5% acetic acid to stop the reaction. Bands in the lane, with full length and catalytic inactive mutants (K155M and T286V) of NLK precipitates, were excised and subjected to in-gel digestion, as previously described [31]. Concisely: After destaining and rehydration with neat acetonitrile, the samples were proteolyzed overnight, using porcine modified trypsin (Promega). The generated peptides were analyzed by peptide mass fingerprinting, using a matrix-assisted laser desorption/ionization time-of-flight mass spectrometer (Ultraflex tandem time-of-flight, Bruker Daltonics). The instrument settings were optimized for analytes with masses from 600 to 4500 Da, and α-cyano-4-hydroxycinnamic acid was used as matrix. The peptide mass list was used for protein identification when searching sequence databases, and Pro-Found at the PROWL web site was used as search engine.

### Immunohistochemistry analysis

The immunohistochemical staining was performed in an automated immunostainer (DAKO Auotstainer plus, Dakocytomation, Denmark). The tissue sections were incubated with the NLK antibody at a dilution of 1:200 (AbCam ab26050), and were subsequently treated with the Dako EnVisionTM FLEX antigen retrieval EDTA buffer (pH 9), using DAKO PT Link module (PT Link, Dakocytomation, Denmark), in accordance with the manufacturer's instructions. A breast pathologist, who at the time of examination was withheld information on diagnosis and other clinicopathological data, evaluated the antibody staining.

### Apoptosis and cell proliferation

Cells were grown on six-well plates, harvested at 24, 48, and 72 hours post-transfection, and counted using Burkers chamber, or Countess Automated Cell Counter (Invitrogen). For apoptosis analyses, the cells were fixed in PFA on coverslips, and stained with Vindelöv solution, containing propidium iodide. After washing, the coverslips were mounted onto glass slides, and examined by fluorescence microscopy. Cells were scored for apoptosis, based on nuclear morphology. Apoptosis was furthermore evaluated, using NucleoCounter NC-3000 (Chemometec), in conformity with the DNA fragmentation assay. Concisely: Transfected and non-transfected cells (controls) were grown on 6-well plates. Cells were harvested by trypsinization, and the trypsinized cells were pooled with the cells floating in the medium. After a short centrifugation, the supernatant was removed and the precipitated cells were washed once with PBS. After a second centrifugation, the cells were resuspended in a small volume of PBS, and the single-cell suspensions were added to 70% ethanol for fixation. Samples were vortexed and stored for 12–24 hours at −20°C. Ethanol-suspended cells were centrifuged and the ethanol carefully decanted. Cells were washed once with PBS and then resuspended with NucleoCounter Solution 3 (1 µg/ml DAPI, 0.1% Triton X-100 in PBS), followed by incubation for 5 minutes at 37°C. Samples of 10 µl volume were loaded into a slide chamber (NC-slide A8), and the DNA Fragmentation protocol was employed according to manufactures instructions (Chemometec).

### GST pull-down

GST pull-down assay was performed incubating 500 ng His-HSP27 (Calbiochem) with 500 ng of GST-NLK recombinant protein (Abnova) in 400 µl binding buffer (20 mM HEPES, pH 7.5, 0.01% NP-40, 0,01% BSA, 100 mM KCl, 5 mM DTT) with agitation for 3 hour at 4°C. Thereafter 30 µl µMACS anti-GST MicroBeads (Miltenyi Biotec) was added and following 30 minutes incubation at 4°C, protein separation was performed on a μ Column in a magnetic field of µMACS Separator (Miltenyi Biotec) according to manufacturer's protocol. GST pull-downs were analyzed with SDS-PAGE and Western blotting.

### In vitro kinase assay

To investigate NLK activity, we examined the phosphorylation of LEF1 in MCF10A, MCF7 and MDA231 cells by performing immunoprecipitation using anti-NLK antibody. Immunoprecipitates were purified by washing two times with lysis buffer containing high salt (0.625 M NaCl), three times with PBS containing high salt (0.625 M NaCl), and two times with PBS. The pellet were incubated with 0.5 µg LEF1 (Novus Biologicals H00051176-P01) and 1 mM ATP in 40 µl of kinase buffer (10 mM MgCl_2_, 10 mM HEPES (pH 7.4), and 1 mM DTT) for 30 minutes at 30°C.

### Statistical analysis

Data are presented as mean ± s.e.m. Statistical comparisons were assessed by ANOVA, or by Student's t-test (p<0.05).

## Results

### Endogenous NLK is localized to the nuclei of breast cancer cells

**Figure 1:**
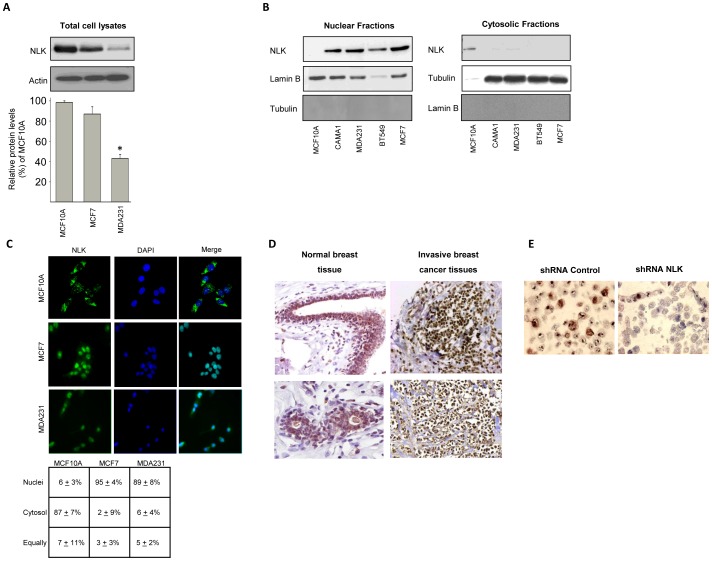
Endogenous NLK is localized in the nuclei of breast cancer cells and breast tumor tissue. (A) The levels of NLK in total cell lysates of MCF10A, MCF7, and MDA231 (upper panel). Densitometric analyses of the total levels of NLK in MCF7, and MDA231 cell lines (Lower panel) relative to NLK protein expression levels in MCF10A cells as percentage (mean ± s.e.m., p<0.05, n = 6). Densitometric analysis of NLK immunoblots was performed in the linear range of detection, and absolute values were then normalized to actin. (B) Isolation of nuclear (left panel) and cytosolic (right panel) fractions by a subcellular fractionation assay followed by a Western blot analysis, using NLK, tubulin or lamin B antibodies. (C) Cellular distribution of NLK in MCF10A, MCF7 and MDA231 cells. Cells were grown on glass coverslips in 6-well plates. After fixation, cells were permeabilized with a 0.25% Triton X-100 solution. Cells were further blocked with 1% BSA, and probed with antibodies against NLK. Nuclei were counter-stained with DAPI. Right panel shows percentage of cells harboring NLK localization in the nuclei, cytosol, or distributed equally between the nucleus and the cytosol. Six to eight microscopic fields of four independent staining were analyzed. (D) Immunohistochemical analyses of subcellular localization of NLK in two normal human breast tissues and in two invasive breast cancer tissues. (E) MCF7 cells were transfected with 40 nM control or NLK siRNA for 24 hours and trypsinized before fixation with 4% formaldehyde for 30 minutes and staining with Meyers's haematoxylin for 5 min. Cells were subsequently centrifuged at 1400 rpm for 5 min and cell pellet were resuspended in 70% ethanol overnight. Cells pellets were dehydrated in graded ethanol series, embedded in paraffin and stained using NLK antibodies.

The total levels of NLK in human breast cancer cells (MDA231 and MCF7) and non-cancerous human breast epithelial cells (MCF10A) revealed that NLK was significantly down-regulated in MDA231 but not in MCF7 compared to MCF10A cells (Figure 1A). In terms of NLK localization by subcellular fractionation, reduced NLK expression was observed in the cytosolic fractions of breast cancer cell lines, compared with non-cancerous human breast epithelial cells MCF10A (Figure 1B). Confocal microscopy and statistical analysis on the endogenous distribution of NLK disclosed that NLK was localized specifically in the nuclei of cancer cells, whereas in MCF10A cells, we observed a cytosolic predominance (Figure 1C), suggesting that NLK localization differs between breast cancer cells and non-cancerous cells. Immunohistochemical staining of human breast tissue, cancerous and normal, showed NLK to be localized in the nuclei of cancer cells, while normal breast tissue contained cytosolic NLK (Figure 1D). Furthermore, by treating MCF7 cells with siRNA against NLK, we could show NLK antibody specificity for immunohistochemistry (Figure 1E).

### Prevention of NLK autophosphorylation directs NLK to the cell nucleus

**Figure 2:**
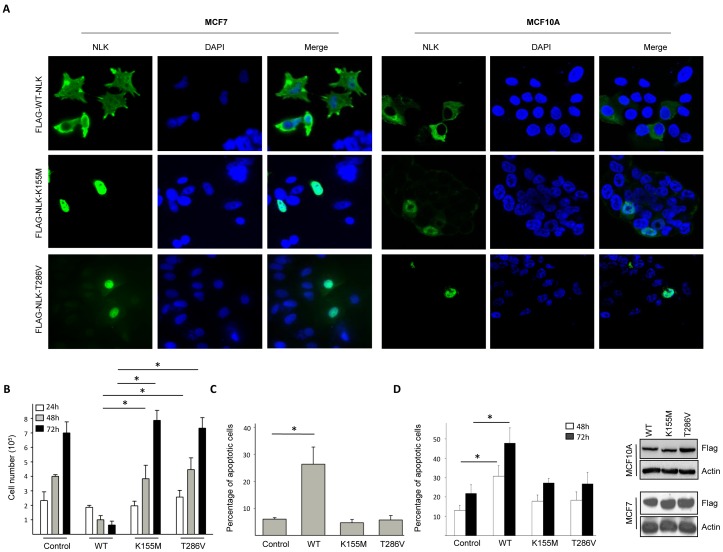
Cytosolic localization of NLK induces cell death. (A) MCF7 and MCF10A cells were transfected for 24 hours with vectors encoding FLAG-tag fusions of full-length NLK (WT), or catalytically inactive NLK mutants (K155M and T286V). NLK localization (Green) was visualized by immunofluorescence staining and confocal microscopy. The nuclei of the cells were counter-stained with DAPI (Blue). (B) MCF7 cells were transfected with vectors encoding FLAG-tag fusions of full-length NLK (WT) or catalytically inactive NLK mutants (K155M and T286V), and counted 24, 48, or 72 h post transfection, using a Countess Automated Cell Counter. Data (mean ± s.e.m., p<0.05, n = 4) are expressed as number of viable cells at each time point. (C) MCF7 cells were mock-treated or transfected for 24 hours with WT-, K155M-, or T286V-NLK. The cells were fixed and stained with propidium iodide in order to count the cells displaying an apoptotic nuclear morphology. Data (mean ± s.e.m., p<0.05, n = 3) show the percentage of apoptotic cells in comparison with cells of mock-treated controls. (D) MCF7 cells were mock-treated or transfected for 24 hours with WT-, K155M-, or T286V-NLK. After 48, or 72 hours cells were subjected to DNA fragmentation assay. Data (mean ± s.e.m., p<0.05, n = 3) represent the percent of sub G0 population. Right panels shows the levels of Flag tagged WT-, K155M-, or T286V-NLK overexpressed in MCF10A and MCF7 cells.

**Figure 3:**
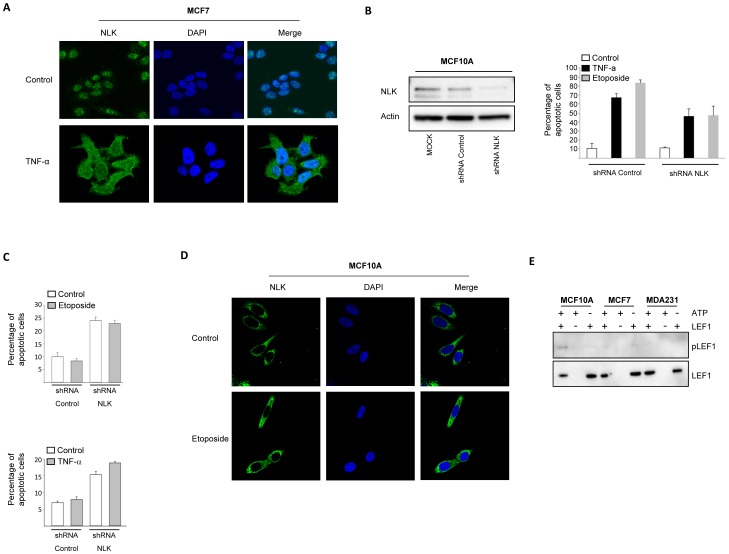
TNF-α and etoposide promotes accumulation of NLK in the cytoplasm. (A) MCF7 breast cancer cells were incubated in the absence or presence of 50 ng TNF-α for 18 hours. The localization of NLK was examined with immunofluorescence followed by confocal microscopy. (B) MCF10A cells were transfected with mock, shRNA targeting NLK, or with a control ShRNA for 24 hours, followed by a Western blot analysis of the total cell lysate, using NLK and Actin (left panel) or subjected to DNA fragmentation assay (right panel, n = 2). Data represent the percent of sub G0 population. (C) MCF7 cells were transfected with shRNA targeting NLK, or with a control ShRNA for 24 hours and treated with etoposide (upper panel, 10 µM for 16 hours) or TNF-α (lower panel, 50 ng TNF-α for 18 hours), followed by DNA fragmentation assay (n = 4). Data represent the percent of sub G0 population. (D) MCF10A cells were grown on glass coverslips in 6-well plates in triplicates. On the following day the cells were treated with DMSO or 100mM of etoposide for 12 hours. After fixation, cells were permeabilized with a 0.25% Triton X-100 solution. Cells were further blocked with 1% BSA, and probed with antibodies against NLK. Nuclei were counter-stained with DAPI. (E) MCF10A, MCF7 and MDA-231 cells were lysed and immunoprecipitated with anti-NLK antibody. Immunoprecipitates were purified by washing two times with lysis buffer containing high salt (0.625M NaCl), three times with PBS containing high salt (0.625 M NaCl), and two times with PBS. The pellet were incubated with 0.5 µg LEF1 and 1 mM ATP in 40 µl of kinase buffer (10 mM MgCl2, 10 mM HEPES (pH 7.4), and 1 mM DTT) for 30 minutes at 30°C. Samples were resolved by SDS-PAGE followed by a Western blot analysis, using phospho-LEF1 and total-LEF1 antibodies.

NLK mutants Lys 155 (K155M) and Thr 286 (T286V), have been reported to abolish the ability of NLK to autophosphorylate [32]. Investigating whether phosphorylation of NLK is involved in its localization, we analyzed the subcellular distribution of wildtype and two kinase mutants of NLK (K155M and T286V). Overexpression of wildtype NLK in the breast cancer cell lines MCF7, or non-cancerous human breast epithelial cells MCF10A, displayed a dominant cytosolic accumulation in all cells (Figure 2A), whereas in the NLK mutants, the distribution was mainly located to the nuclei of MCF7 and MCF10A cells (Figure 2A). To determine whether loss of NLK in the cytosol would have an oncogenic effect, we examined proliferation and survival of cancer cells in the presence of wildtype or catalytically inactive NLK mutants. MCF7 cells, transiently expressing wildtype NLK, reduced the proliferation rate and the cell number at 24, 48, and 72 hours post-transfection, in comparison with non-transfected cells (Figure 2B). In contrast, no differences in cell number were observed when MCF7 cells were transfected with NLK-K155M and NLK-T286V (Figure 2B). To establish whether cell death was responsible for the reduced cell number observed in the transfected wildtype cells, we performed two different apoptosis assays (Figure 2C and 2D). The number of apoptotic cells in the wildtype but not in catalytically inactive NLK-expressing cells was increased when compared to non-transfected controls (Figure 2C and 2D). Furthermore, induction of apoptosis by TNF-α promoted accumulation of NLK in the cytoplasm in MCF7 cells (Figure 3A), whereas NLK shRNA-treated MCF10A cells were less sensitive for TNF-α or etoposide-induced apoptosis compared to control cells (Figure 3B). However, down-regulation of NLK in MCF7 cells did not affect etoposide or TNF-α-induced apoptosis (Figure 3C), suggesting that reduction of the NLK levels is not essential for survival of MCF7 cells. In addition, treatment of MCF10A cells with etoposide did not affect the localized distribution of NLK (Figure 3D). Next, we immunoprecipitated NLK from MCF10A, MCF7 and MDA231 cells and mixed that with purified LEF1 as substrate and could show that endogenous NLK expressed in MCF10A cells induces LEF1 phosphorylation at Thr155 (Figure 3E). These results suggest that NLK expressed in MCF7 and MDA231 cells is catalytically inactive.

### Heat-shock protein 27 recognizes and binds to catalytically inactive NLK in the nucleus

**Figure 4:**
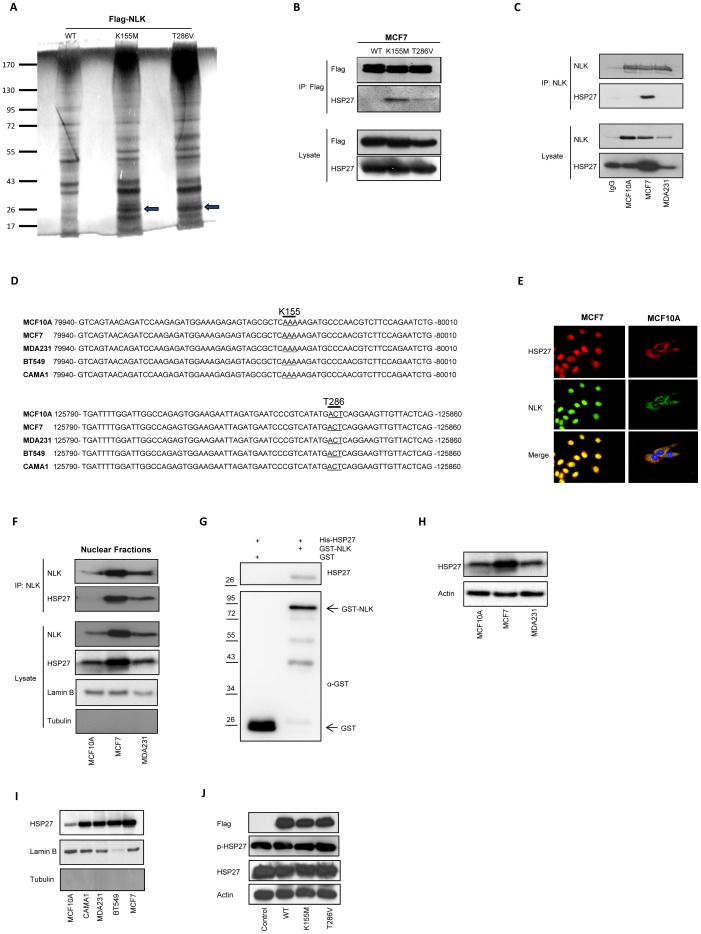
Heat-shock protein 27 binds to NLK in the nucleus. (A) MCF7 cells, transfected with FLAG-tagged WT-, K155M-, or T286V-NLK plasmids, were harvested for immunoprecipitation 24 hours post-transfection, using FLAG antibody. The protein lysates were separated by SDS-PAGE, and the protein bands were cut and processed for the MALDI-TOF analysis. Arrows indicate a specific HSP27 band in NLK-K155M as well as NLK-T286V transfected cells. (B) Immunoprecipitation of FLAG in total cell lysates from MCF7 cells transfected with wild type (WT) and catalytic inactive mutants (K155M and T286V) of NLK, followed by a Western blot, using FLAG or HSP27 antibodies. (C) Endogenous immunoprecipitation of NLK in total cell lysates from MCF10A, MCF7, and MDA231 cells, and a Western blot, using NLK or HSP27 antibodies. As control we used anti-rabbit negative control IgG for immunoprecipitation in total cell lysates from MCF10A cells. (D) DNA sequencing and alignment of NLK gene using MCF10A, MCF7, MDA231, BT549 and CAMA1 cell lines. (E) The cellular distribution of NLK and HSP27 in MCF10A and MCF7 cells, using immunofluorescence staining and confocal microscopy. (F) Endogenous immunoprecipitation of NLK in nuclear fractions of MCF10A, MCF7, and MDA231 cells, and a Western blot, using NLK or HSP27 antibodies. The lysate from nuclear fraction were followed by a Western blot analysis using lamin B or tubulin antibodies. (G) Recombinant GST-NLK and His-HSP27 were incubated in a binding buffer and thereafter GST was pulled down with GSH-coupled sepharose. Pull-downs were analyzed with Western blot using GST or HSP-27 antibodies. (H) The levels of HSP27 in total cell lysates of MCF10A, MCF7, and MDA231 cells. (I) Isolation of nuclei by a subcellular fractionation assay, followed by a Western blot analysis, using HSP27, tubulin, or lamin B antibodies. (J) The levels of HSP27 and phosphorylated HSP27 in total cell lysates of MCF7 cells transfected with wild type (WT) and catalytic inactive mutants (K155M and T286V) of NLK, followed by a Western blot.

Seeking an explanation for NLK inactivation in cancer cells, we performed a MALDI-TOF mass spectrometry analysis. Immunoprecipitation of wildtype NLK and two NLK mutants (NLK-K155M and NLK-T286V) in MCF7 transfected cells, disclosed heat-shock protein 27 (HSP27; Figure 4A). This novel binding partner was detected in cells transfected with NLK-K155M and NLK-T286V, but not in cells overexpressing wildtype NLK (Figure 4A). These results were confirmed by an immunoprecipitation analysis in MCF7 cells, transfected with wildtype, NLK-K155M, or NLK-T286V expression plasmids (Figure 4B). Endogenous immunoprecipitation of NLK demonstrated a clear interaction with HSP27 in MCF7 cells, but not in MCF10A cells (Figure 4C). Interaction not being observed between NLK and HSP27 in MDA231 cells might be due to the low level of NLK expression in the total cell lysate (Figure 4C). Since we found no NLK mutations (K155 or T286) in four breast cancer cell lines and non-cancerous MCF10A cells, this indicates that these mutations alone are not responsible for NLK association to HSP27 (Figure 4D). Furthermore, we detected co-localization and endogenous association between NLK and HSP27 in the nuclei of MCF7 cells, but not in MCF10A cells (Figures 4E and 4F). To investigate whether NLK associates directly with HSP27, we performed an in vitro pull-down assay and found a direct interaction between NLK and HSP27 (Fig. 4G). HSP27 is ubiquitously expressed in different tissues and cells, and localizes under normal conditions to the cytosol. However, HSP27 is able to translocate into the nucleus in response to heat shock or stress [33], [34]. Although we could observe an increase in the levels HSP27 in MCF7 cells compared with MCF10A or MDA231 cells (Figure 4H), we noted that HSP27 localization paralleled that of NLK (Figure 4I and Figure 1B). The level of HSP27 in the nuclear fraction of MCF10A cells was distinctly lower than the HSP27 levels in different breast cancer cell lines (Figure 4I). Since we could not observe any differences in the phosphorylation status of HSP27 (serine 82) in cells transfected with wildtype NLK, NLK-K155M and NLK-T286V expression plasmids, this suggest that NLK is unable to increase the levels of phosphorylated HSP27 at serine 82 (Figure 4J). However, we cannot rule out the possibility that NLK phosphorylate HSP27 at another site beside serine 82. These results together suggest that in an inactive form, NLK is able to associate with HSP27 in the nuclei.

### Down-regulation of HSP27 releases NLK to the cytosol, which induces further cell death in cancer cells

**Figure 5:**
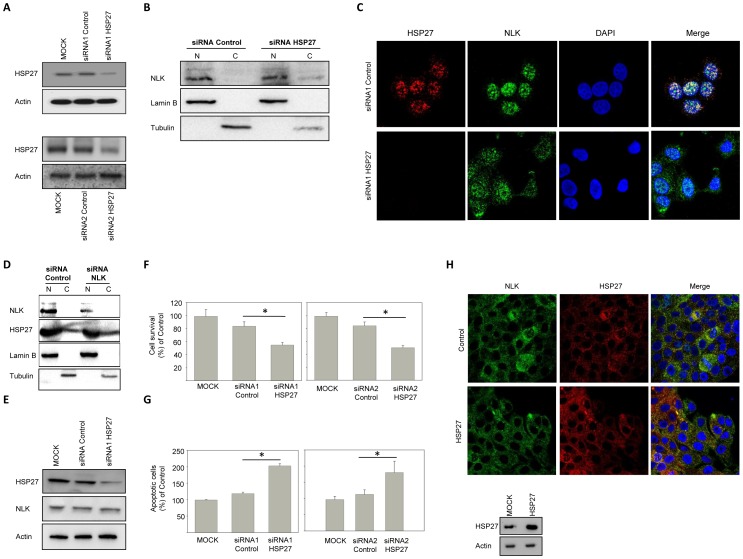
Release of NLK to the cytosol by down-regulation of HSP27 augments apoptosis induction. (A) MCF7 cells were mock-treated, or transfected with two different siRNAs targeting HSP27 (siRNA1 HSP27 or siRNA2 HSP27) or with a control oligonucleotide, twice for 24 hours, followed by a Western blot analysis, using HSP27 and Actin. (B) MCF7 cells were transfected with siRNAs targeting HSP27 or with a control oligonucleotide, twice for 24 hours. Nuclear (N) and cytosolic (C) fraction from total cell lysate was fractionated and followed by a Western blot analysis, using NLK, lamin B, or tubulin antibodies. (C) The cellular distribution of NLK in MCF7 cells transfected two times within 48 hours with siRNA oligos against HSP27. After fixation, cells were permeabilized with 0.25% Triton X-100 solution, after which they were blocked with 1% BSA, and subsequently probed with antibodies against NLK (1:100) and HSP27 (1:100). After washing the coverslips, fluorescent antibodies: Alexa fluor 488 goat anti Rabbit (1:1000) or Alexa fluor 568 Donkey anti Goat (1:1000) were applied and nuclei were stained with DAPI. (D) MCF7 cells were transfected with siRNAs targeting NLK or with a control oligonucleotide, twice for 24 hours. Nuclear (N) and cytosolic (C) fraction from total cell lysate was fractionated and followed by a Western blot analysis, using NLK, HSP27, tubulin, or lamin B antibodies. (E) Endogenous levels of NLK and HSP27 in total cell lysates from MCF7 cells, mock-treated, or transfected with siRNA against HSP27 or with a control oligonucleotide, twice for 24 hours. (F) MCF7 cells, transfected with siRNA, were evaluated 24 hours after secondary transfections, using the NucleoCounter cell viability assay. Data (mean ± s.e.m., p<0.05, n = 3) are presented as the amount of viable cells in comparison with mock-transfected cells. (G) MCF7 cells, transfected with siRNA, were fixed and stained with propidium iodide, 24 hours after secondary transfections. Cells were visualized by fluorescence microscopy, and scored for apoptotic nuclear morphology. Data (mean ± s.e.m., p<0.05, n = 3) show the amount of apoptotic cells in comparison with mock-transfected cells. (H) The cellular distribution of NLK in MCF10A transfected with HSP27 for 24 hours using immunofluorescence staining and confocal microscopy (upper panel). Lower panel shows the levels of HSP27 in transfected MCF10A cells.

To test the hypothesis that HSP27 is responsible for sustained nuclear localization of NLK, we reduced the level of HSP27 in cancer cells, by using two different siRNA oligos (Figure 5A), and investigated NLK localization. HSP27 depletion in MCF7 cells prompted re-localization of NLK from the nucleus to the cytoplasm and reduced the levels of NLK in the nucleus (Figure 5B and 5C). Down-regulation of NLK did not affect the localization of HSP27 in MCF7 cells, implying that the levels of NLK in MCF7 cells is not essential for localization of HSP27 (Figure 5D). Furthermore, we did not, observe any differences in total NLK levels after treating the cells with siRNA for a knockdown of HSP27 (Figure 5E). To explore whether NLK-mediated apoptosis is dependent upon the association with HSP27, we down-regulated HSP27 in MCF7 cells, and subjected the cells to survival and apoptosis assays. As expected, reduced level of HSP27 in MCF7 cells diminished cell survival (Figure 5F), and induced apoptosis (Figure 5G). In contrast, HSP27 overexpression in MCF10A cells was unable to re-localizes NLK to the nucleus (Figure 5H). These results suggest that NLK is captured by HSP27 in the nucleus, whereas depletion of HSP27 releases NLK to the cytosol, which induces further cell death in cancer cells.

## Discussion

The serine/threonine kinase NLK is an atypical MAP kinase, able to phosphorylate several target proteins in cytosol and nucleus, and to regulate different signaling pathways. Evidence for endogenous subcellular localization of NLK, and for the mechanism regulating its nuclear import, is limited. We found in the present study that endogenous NLK was localized predominantly in the nuclei of breast cancer cells, in contrast to a cytosolic localization in non-cancerous cells. The endogenous nuclear localization of NLK in breast cancer cells did not change by cancer cells being transfected with NLK mutants, which prevent NLK activation. We further confirmed that overexpression of wildtype NLK is localized predominantly in the cytosol. Contrasting these results, in prostate cancer cells, endogenous NLK were localized in the nucleus (LNCaP), but the wildtype NLK expression in LNCaP cells also directed NLK to the nucleus [35]. This discrepancy may be explained by cell type specificity as well as androgen-mediated receptor activation, both proved to be essential for NLK localization in prostate cancer cells. Furthermore, our data agree with previous observations of NLK kinase negative mutant being accumulated in the nucleus upon overexpression [29], [36].

The tumor suppressor function of NLK was recently established. For example, overexpression of wildtype NLK in colon carcinoma cells, boosted programmed cell death through phosphorylation of CBP, and hence, suppression of the transcriptional activity of AP-1, Smad, and p53, all of which use CBP as a co-activator [4], [26]. In prostate cancer, overexpression of NLK induced apoptosis in AR-expressing prostate cancer cells, but not in AR-negative cells [27]. To determine whether NLK localization regulates cell survival in breast cancer cells, wildtype or phospho-mutants of NLK were overexpressed in these cells. Wildtype NLK reduced proliferation rate and cell survival, while NLK mutants had no significant effect, suggesting that in contrast to inactive nuclear localized NLK, cytosolic localization of NLK triggers cell death. This was evident by stimulation of breast cancer cells with TNF-α, which promoted re-localization and accumulation of NLK in the cytoplasm. In contrast to the endogenous NLK expressed in MCF10A, which was phosphorylated and catalytically active, NLK expressed in breast cancer cells showed to be unphosphorylated and catalytically inactive.

To find an explanation for the inactivation of NLK in cancer cells, we explored whether any putative interaction partners for NLK might hold NLK in an inactive form in the nucleus of cancer cells. Cells were thus transfected with wildtype and phospho-mutant NLK prior to MALDI-TOF mass spectrometry analysis. HSP27 was identified as a novel binding partner for the inactive form of NLK. Endogenous immunoprecipitation of NLK in MCF7 cells, and cells transfected with the phospho-mutant form of NLK, both confirmed this finding. As expected, we were unable to co-precipitate HSP27 in non-cancerous MCF10A cells, or when wildtype NLK was overexpressed. HSP27 is a ubiquitously expressed protein, able to translocate into the nucleus in response to heat shock or stress [33], [34]. We could also show that NLK is unable to phosphorylate HSP27 at serine 82 in cells transfected with wildtype NLK, but we cannot rule out the possibility that NLK phosphorylate HSP27 at another site beside serine 82.

In breast cancer cells, we noted that HSP27 localization was similar to NLK localization, and by confocal imaging, we confirmed co-localization of these two proteins in cancer cells. Our results suggest that HSP27 recognizes and binds to the non-phosphorylated form of NLK, which is exclusively located in the nuclei of cancer cells. Since we found no NLK mutations at lysine 155 or threonine 286 in breast cancer cell lines, this indicates that these mutations alone are not responsible for NLK association to HSP27. Further studies are needed to define how inactive mutant of NLK can associate with HSP27 in breast cancer cells.

HSP27 functions mainly as a chaperone in protein folding, but is also implicated in cytoskeleton rearrangement, cell movement, cell survival, and tumor progression [37]. In general, the aberrant expression of HSP27 is associated with aggressive tumor behavior, increased resistance to chemotherapy, and poor prognosis for the patient [38], [39]. More specifically, high levels of HSP27 have been observed in breast cancer cells, in comparison with normal cells [40]–[42].

To evaluate whether the HSP27 association with NLK in breast cancer cells may affect NLK localization, we treated cancer cells with siRNA against HSP27, and found that HSP27 depletion in MCF7 cells elicited NLK re-localization from the nucleus to the cytosol. In contrast to this finding, HSP27 overexpression in non-cancerous epithelial cells was unable to re-localizes NLK to the nucleus. The treatment of breast cancer cells with siRNA against HSP27, to explore whether the HSP27 association with NLK regulates NLK-mediated cell death, reduced cell survival, and increased the rate of cell apoptosis, suggesting that an inactive form of NLK is captured by HSP27 in the nucleus, while depletion of HSP27 releases NLK to the cytosol, which consequently induces further cell death in breast cancer cells. Since, down-regulation of NLK in MCF7 cells did not affect cell survival, this suggest that reduction of the NLK levels is not important for cell apoptosis instead NLK translocation from the nuclei to the cytoplasm, which execute cell death. We could also observe that NLK overexpression-induced apoptosis was reduced by HSP27 overexpression. Further investigations are required to identify the signaling pathway, enabling cytosolic NLK to induce apoptosis.

In conclusion, our findings unveil a novel mechanism, by which HSP27 recognizes NLK in breast cancer cells and prevents NLK-mediated cell apoptosis.
